# Tiny-Machine-Learning-Based Supply Canal Surface Condition Monitoring

**DOI:** 10.3390/s24134124

**Published:** 2024-06-25

**Authors:** Chengjie Huang, Xinjuan Sun, Yuxuan Zhang

**Affiliations:** 1 School of Electronic Engineering, North China University of Water Resources and Electric Power, Jinshui East Road No. 136, Zhengzhou 450046, China; 2 Department of Computer and Electrical Engineering, Mid Sweden University, 85170 Sundsvall, Sweden

**Keywords:** tiny machine learning (TinyML), structural health monitoring (SHM), damage classification, embedded systems, convolutional neural networks (CNNs), water supply canals

## Abstract

The South-to-North Water Diversion Project in China is an extensive inter-basin water transfer project, for which ensuring the safe operation and maintenance of infrastructure poses a fundamental challenge. In this context, structural health monitoring is crucial for the safe and efficient operation of hydraulic infrastructure. Currently, most health monitoring systems for hydraulic infrastructure rely on commercial software or algorithms that only run on desktop computers. This study developed for the first time a lightweight convolutional neural network (CNN) model specifically for early detection of structural damage in water supply canals and deployed it as a tiny machine learning (TinyML) application on a low-power microcontroller unit (MCU). The model uses damage images of the supply canals that we collected as input and the damage types as output. With data augmentation techniques to enhance the training dataset, the deployed model is only 7.57 KB in size and demonstrates an accuracy of 94.17 ± 1.67% and a precision of 94.47 ± 1.46%, outperforming other commonly used CNN models in terms of performance and energy efficiency. Moreover, each inference consumes only 5610.18 μJ of energy, allowing a standard 225 mAh button cell to run continuously for nearly 11 years and perform approximately 4,945,055 inferences. This research not only confirms the feasibility of deploying real-time supply canal surface condition monitoring on low-power, resource-constrained devices but also provides practical technical solutions for improving infrastructure security.

## 1. Introduction

The Middle Route of the South-to-North Water Diversion Project in China is a significant large-scale hydraulic infrastructure endeavor implemented to optimize the spatiotemporal distribution of water resources, which is essential for future Chinese sustainable development [1]. The project covers a wide area with variable geographic conditions, which means that the canal will be affected by a variety of factors such as precipitation, temperature, soil quality, and settlement, resulting in complex cross-section changes. These changes frequently pose risks of structural instability. Visible cracks on the surfaces of supply canals serve as direct indicators of internal geological changes. If these cracks are not detected and repaired in time, they could lead to severe consequences [2,3]. First, cracks in the supply canal lead to significant water seepage and loss during transportation [4]. Over time, as water erosion within the canal progresses, these cracks may expand, potentially triggering major safety incidents and affecting normal operations. Since the completion of the project, crack detection has predominantly relied on regular manual inspections, which are not only costly but are also prone to inevitable errors such as misses, false positives, and redundancies [5,6,7].

The advancement of remote sensing technology has provided a robust data source for classifying damage in the water supply canals of the project using remote sensing imagery [8]. Moreover, the development of artificial intelligence and information technology, including the introduction of machine learning, has revolutionized the field of infrastructure health monitoring [9,10]. These technologies enable systems to learn from data and enhance performance, significantly improving the efficiency of structural damage detection. Convolutional neural networks (CNNs) are a subset of deep learning techniques that facilitate more accurate and scalable damage detection systems, enhancing comprehensive autonomous assessment and prediction of structural damage [11,12]. The effectiveness of current CNNs in the field of structural health monitoring underscores the efficiency of deep learning techniques for analyzing complex data [13,14]. Recent studies have confirmed the role of CNNs and related technologies for efficiently detecting and classifying crack damage [15,16]. Study [17] proposed an integrated approach using CNNs to analyze and classify structural damage in masonry by utilizing traditional CNN architectures like AlexNet and GoogLeNet and achieved an accuracy of 94.3% for damage classification. In [18], the accuracy of damage classification is evaluated using various CNN architectures, such as Inception V2 and MobileNet’s Single-Shot MultiBox Detector (SSD), which are deployed on GPU servers and smartphones. In [19], a vision-based method using deep CNNs is proposed for detecting concrete cracks in infrastructure; the CNN is trained on 40,000 images and achieved 98% accuracy, demonstrating the potential of deep learning for damage detection in building infrastructure inspection, particularly for automatic detection and classification. Study [20] explored the capacity of CNNs to classify and detect structural damage in steel and aluminum building models. It found that CNNs can efficiently classify damage even with limited sensor coverage, demonstrating their capacity to enhance the accuracy and efficiency of structural health detection. Another study [21] utilized CNNs to address the limitations of manual visual inspections of bridges by employing a dataset of 6900 images to classify defects with a 97.13% accuracy rate, demonstrating the potential of deep learning technology to provide precise defect localization and automated detection.

Despite the notable success of these methods at enhancing accuracy, an overemphasis on such enhancements often leads to high demands on computational resources, overshadowing the essential need for sustainable real-time data processing and edge deployment [22]. The emergence of tiny machine learning (TinyML) provides a new solution in the domain of infrastructure health monitoring [23] and makes it feasible to execute deep learning algorithms at the edge [24]. Processing sensor data and running inference at the edge significantly reduces the energy needed to transmit sensor data and communications [23]. Currently, TinyML has been applied in several fields, including medical, environmental monitoring, agricultural technology, and smart cities [25,26,27,28,29]. In smart building infrastructure, a research team developed a water leakage detection system using TinyML technology that captures real-time data from sensors through edge computing and deploys the EfficientNet model to analyze it. This system achieved an accuracy rate of 97.45% in water leakage detection, with an F1-score of 97.63%, demonstrating its efficient application in building management [30]. In [31], other researchers utilized an unsupervised TinyML framework and IoT (Internet of Things) technology to analyze urban noise data with a long- and short-term memory autoencoder to effectively detect urban noise anomalies. This model achieved an accuracy of 99.99% and an F1-score of 99.34%, demonstrating TinyML’s strong potential in real-time urban noise monitoring. Ref. [32] introduces a smart weather measurement system based on TinyML that deploys multiple heterogeneous nodes and controllers equipped with AI algorithms in wind turbine areas. This system provides real-time weather data at a low cost and is a pioneering innovation in environmental monitoring and smart agriculture. In [33], the researchers achieved 75–95% execution accuracy by training a multi-layer long short-term memory (LSTM) model on a finger-worn ring device. This demonstrates the great potential for real-time analytics using TinyML in wearable devices, particularly for health detection and gesture recognition. Miguel de Prado and his team utilized TinyML technology to implement autonomous driving in a low-power automated micro-vehicle. By combining compact TinyML and GAP8 ultra-low-power RISC-V microcontroller units (MCUs), they achieved high-precision predictions under varying light conditions, attaining an accuracy of 97.4%. This showcases great potential for deploying autonomous mini-vehicles [34]. Therefore, TinyML techniques can be applied to the structural health monitoring of water infrastructure.

The main contributions of this paper are as follows:

- We designed and evaluated a custom lightweight CNN architecture for remote sensing images and compared its performance with that of common CNN models.
- For the first time, we applied the proposed CNN model to classify cracks in supply water canals and deployed it on low-power, resource-constrained MCUs, and we also explored the deployability of other CNN models on MCUs.
- In addition to accuracy and model size, we also comprehensively compared deployable CNN models in terms of RAM, flash usage, energy consumption, and inference time, providing a feasibility exploration for continuous online health monitoring of hydraulic infrastructure based on remote sensing imagery.

**Figure 1:**
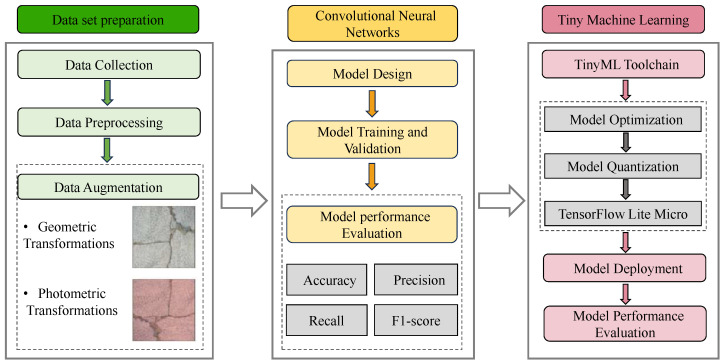
Workflow of the paper.

The overall flow of the work in this paper is shown in Figure 1. The flow is consistent with the article structure of this paper, which is divided into the following sections: Section 2 introduces the data collection and preprocessing methodologies. Section 3 details the proposed lightweight CNN model and its design process, development environment, and the tools used for training, validation, and testing, along with comparisons to previous related models and the outcomes of the proposed model, such as parameters, accuracy, and training curves. Section 4 describes the TinyML toolchain, the workflow used, and the specifications of the MCU used for model deployment, including the deployment process and post-deployment performance metrics such as accuracy, RAM and flash usage, inference times, and energy consumption estimates. Finally, Section 5 presents the conclusions of this study and future work directions.

## 2. Datasets

This section introduces, in detail, the collection of image datasets, data preprocessing, and data augmentation methods for small datasets.

### 2.1. Data Collection

**Figure 2:**
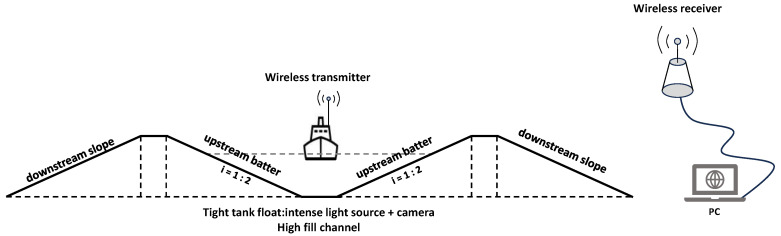
Water supply channel data acquisition device.

**Figure 3:**
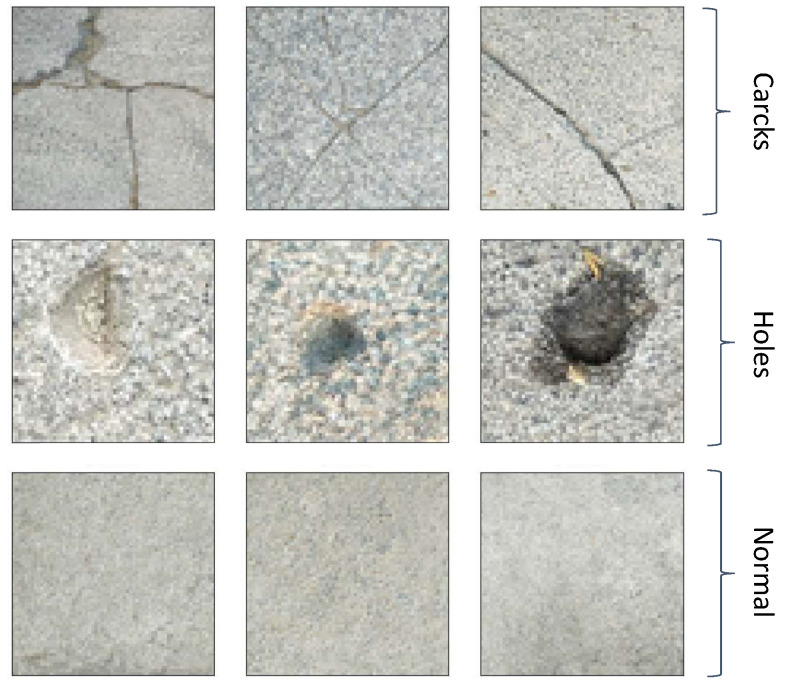
Original image examples.

In this paper, we construct an experimental model of the supply canal for the South-to-North Water Diversion Middle Line project, which features a backwater slope and a water-facing slope with a slope ratio of 1:2 [35]. A high-definition (HD) video camera is mounted in a removable suspension silo on the device, allowing flexible positioning during experiments to ensure comprehensive coverage of the water-facing slope’s surface. The camera captures images of the recharge channel on the water-facing side of the slope, detailing normal conditions as well as cracks and holes, and transmits these data to a PC terminal via a wireless network for real-time viewing and analysis. The camera must operate effectively under diverse lighting conditions to ensure clear image capture both in direct daylight and in shadow. Additionally, the exposure and focus settings need adjustment. Given the limitations of the experimental conditions, this paper concentrates on processing and analyzing the image data of the water supply channel on the water-facing side of the slope, as illustrated in Figure 2. A supply canal is a type of water conservancy structure designed for water transportation and distribution. Typically utilized in large-scale irrigation projects, reservoirs, and hydropower stations, these supply canals are extensively employed in the South-to-North Water Diversion project in China, especially by the Central Route Engineering Agency [36]. They also play an important role in enhancing the utilization efficiency of water resources and promoting regional water circulation. The damage categories of supply canals are classified as normal, cracks, and holes. Holes are defined as visible openings in the cement panels and generally range from 2.5–10 mm in diameter and 10–50 mm in depth and have predominantly blocky or elliptical shapes. Cracks are characterized by their linear features, jagged edges, and intersections and often appear darker than the background in images. If none of the above conditions exist on the waterward slope of the supply canal, it is classified as normal. Sample images from the original dataset are shown in Figure 3.

### 2.2. Data Preprocessing

In this study, to ensure that the data quality and format meet the requirements of deep learning model training, a meticulous preprocessing step is conducted. Before feeding the image data into the deep learning network, a series of data preprocessing operations are performed. The cropped image data are uniformly resized to 64 × 64 pixels to simplify processing; the RGB color format is used, and the images are stored as arrays and normalized to the float32 type [37]. The dataset comprises a total of 270 images that are categorized into three types of damage states: hole-0, crack-1, and normal-2. Each category contains 90 images. Due to the small size of the dataset, to establish an effective training strategy and a robust evaluation basis, we divided the dataset into training, validation, and test sets in a ratio of 4:1:4. Specifically, 120 images are allocated to the training set, 30 to the validation set, and 120 to the test set. The composition of the distribution of the original dataset is shown in Table 1.

### 2.3. Data Augmentation

**Figure 4:**
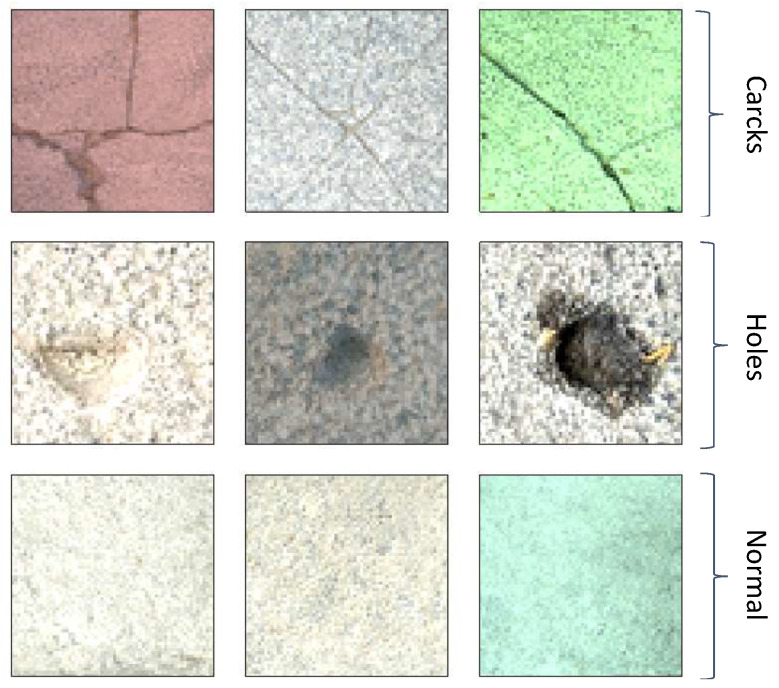
Augmented image examples.

Given the limited size of the dataset, the training dataset is augmented to enhance the model’s generalization ability and mitigate or avoid overfitting. The augmented dataset encourages the model to learn a more robust feature representation, thus improving the stability and reliability of the model [38]. The ImgAug library is utilized for data augmentation in this study [39] and facilitates data augmentation for the training dataset with the aim of enhancing model performance by introducing image diversity. In the image enhancement sequence, various operations are applied to simulate the visual performance of an image under changing environmental conditions. Image translation and rotation operations simulate physical movements or camera angle changes during photography. Gaussian blurring techniques are applied to simulate the blurring effects produced by various lenses focusing [40]. Contrast and brightness are adjusted to simulate shooting under varying lighting conditions, thereby replicating performance in environments ranging from daylight to shadow. Gaussian noise is added to simulate noise interference that may arise from sensors, circuits, or poor lighting conditions during actual shooting [41]. The expanded image is shown in Figure 4. An expanded dataset, sixfold larger than the original, is generated by applying a defined augmentation sequence to each image. Subsequently, the original images are merged with the expanded images to form the final expanded dataset, as shown in Table 2, with the label set also expanded accordingly.

## 3. Convolutional Neural Networks

This section is divided into three subsections, each detailing the architectural design, training process, and performance evaluation of the lightweight CNN model developed for edge devices. Meanwhile, to better evaluate the performance of the proposed lightweight models in structural health monitoring, we compare them with traditional CNN models—including ResNet [42], EfficientNet [43], ShuffleNet [44], MnasNet [45], and MobileNetV2 [46], all of which feature multiple levels of feature extraction mechanisms and have achieved significant results in image classification tasks. This analysis aims to better assess the relative performance of the models proposed in this paper compared to current leading deep learning architectures in terms of efficiency, accuracy, and resource consumption.

### 3.1. Model Design

**Figure 5:**
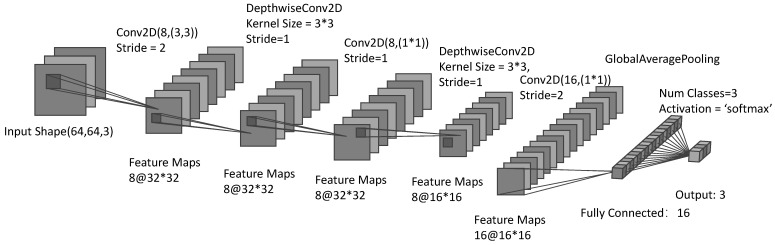
Proposed CNN model structure.

The proposed model in this paper is designed with computational constraints for low-power edge devices in mind and employs a lightweight network framework that reduces the need for computational power and storage, which is essential for real-time system deployments in remote autonomous monitoring. As illustrated in Figure 5, the lightweight model developed in this work, which drew inspiration from MobilenetV2, uses inverted residual blocks that have been optimized to lower complexity. The model employs a multilayer structure for efficient processing of image data [46]. The input layer accepts 64 × 64 RGB images and is followed by a convolutional layer. The convolution operation reduces the size to 32 × 32, increases the count to eight feature maps, and utilizes a normalization layer to normalize the activation values. After spatial filtering with depth-separable convolution, the model gradually totals simple features by increasing the number of layers, transitioning to a more advanced abstract feature representation. Lastly, a global average pooling layer is used to reduce the features to vectors, and triple classification of the image is achieved through an output layer.

The architecture of the proposed model reflects an innovative approach to minimizing the number of parameters and computational cost, enabling more efficient feature extraction and representation under conditions of limited device resources. Incorporating a global average pooling (GAP) layer at the end of the network instead of a fully connected layer significantly reduces the number of parameters, diminishing the model size and computational cost [47]. Additionally, GAP enables the model to accommodate different input sizes and enforce spatial hierarchies in the feature maps to reduce the risk of overfitting. In the design, the number of filters in the initial convolutional layer is intentionally reduced and then increased cautiously to preserve computational resources while still ensuring that the model’s feature extraction performance is optimal. Moreover, the model introduces depth-separable convolution [48], which significantly reduces the computational load and the size of the model, streamlining the detailed filtering of the input. This method does not compromise the quality of feature extraction compared to traditional convolutional layers and offers finer extraction with much lower computational requirements. The design employs the ReLU fractional linear activation function, which introduces fractional linearity into the model, enhancing the learning of complex patterns in the data [49]. The proposed model network architecture differs from traditional CNNs such as ResNet-50, MobilenetV2, and EfficientNet-B0 by explicitly focusing on the constraints of edge computation. While traditional models increase computation to maximize accuracy [50], the proposed model adopts a balanced approach that prioritizes efficiency and effective deployment on edge devices.

### 3.2. Model Training and Validation

**Figure 6:**
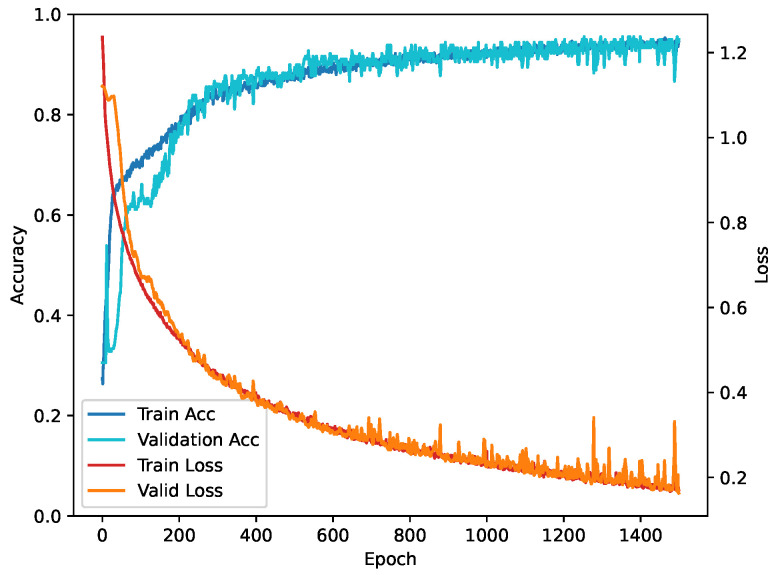
Training and validation loss curves of the proposed model.

In this study, all model training is performed using TensorFlow (TF) [51] on a GeForce GTX 4060 GPU with the CUDA framework. The training utilizes a sparse classification cross-entropy loss function, which is suitable for multi-classification tasks, and optimization utilized the Adam optimizer, which is known for its efficiency at handling sparse gradients. Training occurred over 1500 epochs, with learning rates ranging from 0.01 to 0.00001. The training and validation loss curves are shown in Figure 6. Preliminary experiments demonstrate that while higher learning rates accelerate loss reduction in each epoch, they also introduce instability into the training process. Conversely, lower learning rates result in slower learning progress. After several trials, a learning rate of 0.0001 proved to provide a satisfactory balance between convergence speed and stability [52]. A batch size of 128 is utilized to process the samples in each iteration. For the other CNN models used for comparison, since their structures are relatively complex and prone to overfitting, this paper employs a fine-tuning technique to modify the corresponding layers for adaptation. All the models used for comparison are pre-trained models that have been trained on ImageNet and have already learned rich feature representations. Model training is configured as 20 training cycles with a batch size of 128 and a learning rate of 0.0001.

### 3.3. Model Performance Evaluation

In order to verify the generalization ability and stability of the model proposed in this paper, a five-fold cross-validation approach is utilized. The entire dataset is divided into five non-overlapping subsets, each serving alternately as a validation set while the remaining four are used for training. This approach ensures that each sample is used for both training and validation, thus providing a more comprehensive assessment of model performance. In each fold of the training process, the corresponding training and validation sets are first extracted from the dataset, and then the model is constructed. Four key performance metrics are selected for the evaluation of the CNN model: accuracy, precision, recall, and F1-score. These metrics provide a comprehensive assessment of the effectiveness of the model on different performance dimensions and are essential for ensuring the reliability and utility of the model in real-world applications.

Precision assesses the accuracy of correct predictions and is defined as the ratio of true positives to the sum of true and false positives. This metric is particularly important in structural health detection and reflects the model’s ability to minimize false positives. Precision is calculated using Equation (1) as follows:

(1) $$\mathrm{Precision}=\frac{TP}{TP+FP}$$

Recall indicates the model’s ability to detect all relevant instances and is defined as the ratio of true positives to the sum of true positives and false negatives. This metric is crucial for avoiding missed detection in structural health monitoring, where such oversights can have serious consequences. Recall is calculated using Equation (2) as follows:

(2) $$\mathrm{Recall}=\frac{TP}{TP+FN}$$

The F1-score is the harmonic mean of precision and recall and combines these into a single metric that balances the model’s precision and recall, effectively balancing all positive results and minimizing false positives. The F1-score is calculated using (3) as follows:

(3) $$\mathrm{F1-score}=\frac{2*\mathrm{Precision}*\mathrm{Recall}}{\mathrm{Precision}+\mathrm{Recall}}$$

To accurately assess the performance of our proposed lightweight CNN model on a limited dataset, this study adopts multiple experimental repetitions. We calculate the mean and standard deviation after 10 experimental runs to provide a stable index that can comprehensively reflect the model’s performance, quantify its stability, and ensure the validity and reliability of the results. These metrics allow us to analyze specific strengths and weaknesses from different perspectives and to compare the efficacy of different network architectures and training strategies in a quantifiable way.

The evaluation results for the model are presented in Table 3. ShuffleNetV2 highlights efficient resource utilization, featuring a low number of parameters (1,194,515) and FLOPs (20,652,799), making it one of the models with the lowest complexity and number of parameters. With an accuracy of 94.95 ± 1.84% and an F1-score of 94.75 ± 1.70%, ShuffleNetV2 achieves a balance between performance and size, making it ideal for deployment on resource-constrained devices. The ResNet-50 model, with a high parameter count (23,593,859) and computational intensity (632,909,458 FLOPs), achieves an accuracy of 92.25 ± 1.97%, indicating its suitability for computationally rich environments rather than edge devices. MobileNetV2, with an accuracy of 96.75 ± 1.21% and an F1-score of 96.17 ± 1.67%, boasts a moderate number of parameters and FLOPs (3,572,803 and 52,741,074), demonstrating its computational efficiency and performance suitability for edge device deployment. Both EfficientNet-B0 and MnasNet are designed to balance performance and resource usage. EfficientNet-B0 features 4,053,414 parameters and 66,654,953 FLOPs, while MnasNet includes 5,402,239 parameters and 57,892,038 FLOPs. Both models exhibit closely matched accuracies (94.35 ± 2.19% and 94.85 ± 1.84%) and comparable F1-scores (95.11 ± 1.89% and 94.05 ± 2.10%), making them well-suited for scenarios that require a balance between resource utilization and performance. The dedicated lightweight model, which was optimized for extremely resource-limited environments, features only 803 parameters and 905,618 FLOPs. It achieves impressive performance, with an accuracy of 94.17 ± 1.67% and an F1-score of 94.26 ± 1.94%, representing an optimal balance of performance and resources, which is ideal for edge deployments.

**Figure 7:**
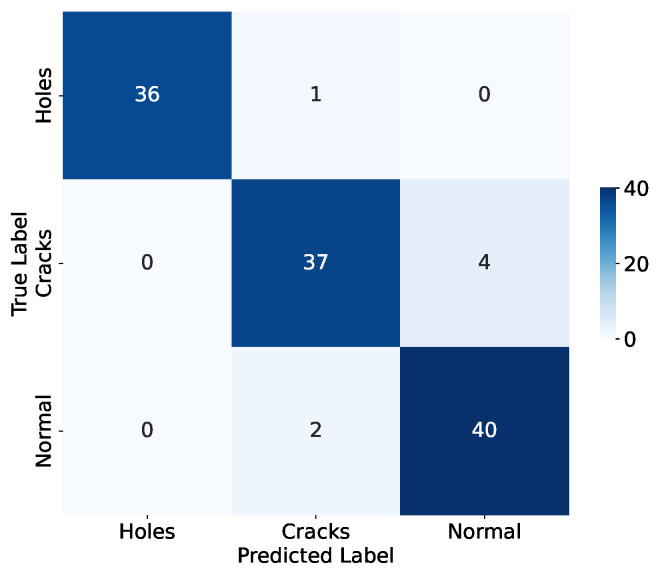
Confusion matrix of proposed model.

The confusion matrix, detailed in Figure 7, records the model’s prediction accuracy for various damage states and aids with performance evaluation. According to the matrix, the model correctly predicted 36 instances of ’holes’, 37 of ’cracks’, and 40 of ’normal’. The primary errors involve the wrong classifications of four ’crack’ instances as ’normal’, pinpointing an area for potential enhancement in the model’s accuracy.

## 4. Tiny Machine Learning

This section details the TinyML implementation process in two main subsections: First, we describe the TinyML toolchain and the development board used in this study. The second subsection addresses the deployment and optimization process of the proposed model; we describe how the model is deployed on an MCU development board and analyze the performance and power consumption of the model.

### 4.1. TinyML Toolchain

TinyML represents a convergence point for embedded ML, hardware, software, and algorithms, which is ideal for the deployment of machine learning models on MCUs and edge devices [53]. These devices typically encounter significant resource constraints. Typically, these models are lightweight versions of traditional architectures, with their sizes reduced through techniques such as pruning [54] and quantization [55]. After training, these models are converted into a low-power device format, such as TensorFlow Lite Micro, to facilitate deployment and inference on edge devices. In this study, the Arduino Nano 33 BLE Sense serves as the experimental platform, and the Arduino IDE is utilized as the development environment. It is powered by an ARM Cortex-M4F processor. The Arduino Nano 33 BLE Sense development board reduces energy consumption via algorithmic optimization and a sleep mode. Table 4 provides key technical specifications for the Arduino Nano 33 BLE Sense.

### 4.2. Model Deployment and Evaluation

This study utilizes TensorFlow Lite Micro (TFLite Micro), which is optimized for microcontrollers and is a framework compatible with the Arduino platform. Deep learning models trained using TensorFlow are transformed into the TensorFlow Lite (TFLite) format for deployment on mobile and embedded devices in order to reduce the model size and computational resource requirements [56]. Subsequently, the model is quantized, which converts 32-bit floating point numbers (float32) to 8-bit integers (uint8) and, thereby, reduces the precision and storage requirements; this adapts the model to resource-constrained embedded environments and significantly reduces the model’s storage footprint and computational resource dependence, facilitating deployment on edge devices. This study employs full-integer quantization to lower the storage requirements and enhance the model’s execution efficiency [57]; further optimization is achieved through the post-training quantization (PTQ) technique, which utilizes a representative dataset for calibrating quantization parameters [58]. Upon completing these steps, the quantized TFLite model is transformed into C language source files through the open-source TFLite Micro tool, enabling operation on the C-language-supported microcontroller nRF52840 and thus enhancing the model’s deployment and operational efficiency on low-power devices. Given the memory limitations of the Arduino Nano 33 BLE Sense platform (only 256 KB RAM), deploying and running a standard CNN model directly on this device is not feasible. Therefore, we employ model quantization and Machine Learning Toolkit (MLTK) evaluation methods to accommodate the hardware’s resource constraints. The MLTK is designed for embedded systems and supports the evaluation of model performance, including accurate measurements of RAM and Flash usage [59].

The model evaluation results are presented in Table 5. The MLTK supports monitoring the use of hardware resources such as Flash and RAM. The performance of various models on specific hardware devices can vary significantly, with measurements of single inference times and power consumption being dependent on the specific hardware environment. Among conventional CNN models, ShuffleNetV2 demonstrates high stability and an F1-score of 93.50 ± 1.88%, and it is economical in terms of flash memory usage (4.70 MB) and RAM usage (167.5 KB), the lowest among traditional CNN models, thus illustrating an optimal balance between processing power and memory requirements. ResNet-50, with an F1-score of 92.56 ± 2.78%, has relatively reliable performance. However, its substantial resource demands exceed the MLTK’s maximum parameterization limits, making specific data on flash and RAM usage unavailable. Typically, it is suited for more complex tasks. The MobileNetV2 model, boasting the highest F1-score of 96.63 ± 1.39%, has significant flash (14.10 MB) and RAM (826.8 KB) requirements, potentially restricting its use on memory-constrained devices. The EfficientNet-B0 and MnasNet models also performed commendably, with F1-scores of 94.42 ± 2.65% and 94.32 ± 2.32%, respectively. Their significant flash (16.00 MB and 21.50 MB, respectively) and RAM (1200.0 KB and 513.50 KB, respectively) demands make them suitable for devices with more available memory. The lightweight model introduced in this study, which is a non-transfer learning model, achieved an F1-score of 94.34 ± 1.64% with minimal flash (0.35 MB) and RAM (96.0 KB) usage, an inference time of 296.94 ms, and energy consumption of 5610.18 microJoules. Out of all the CNN models, only the proposed model can successfully be deployed on MCUs; it strikes a perfect balance between performance and energy efficiency, rendering it an ideal option.

**Figure 8:**
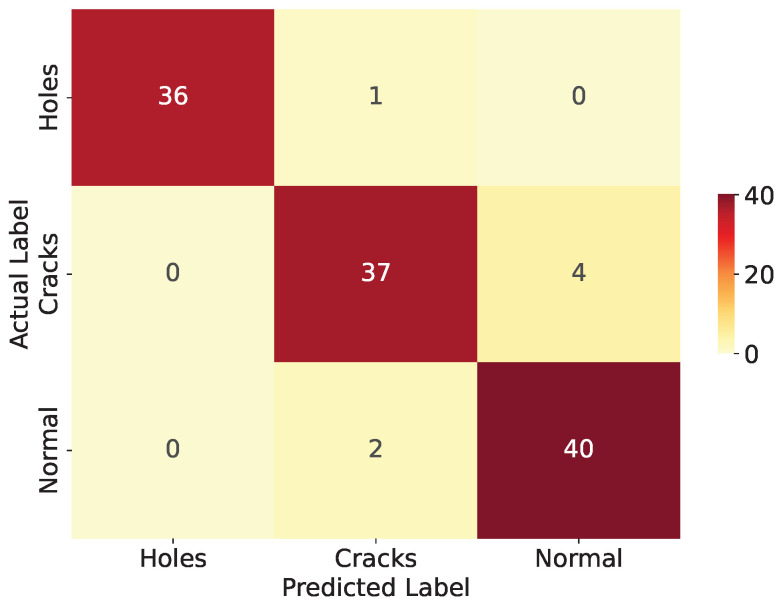
Confusion matrix of deployed proposed model.

After optimization and quantization on the MCU, the model is re-evaluated, and the results, which are depicted in Figure 8, indicate consistent predictive capabilities compared to the CNN model section, confirming that performance is not significantly compromised in constrained environments. This supports the feasibility of deploying deep learning models on edge devices and demonstrates the effectiveness of the TinyML technique at preserving model performance.

(4) $${\mathrm{Energy}}_{\mathrm{Our}\;\mathrm{Model}}=\mathrm{Operating}\;\mathrm{Power}\times \mathrm{Power}\;\mathrm{Consumption}\;\mathrm{per}\;\mathrm{Inference}$$

From the nRF52840 datasheet, as outlined in Table 4, CPU power consumption during operation and model inference times are detailed. For calculations, a typical current of 6.3 mA at a constant voltage of 3 V is used, resulting in a power consumption of approximately 18.9 mW for the nRF52840 microcontroller. Based on the inference time (296.94 ms), the microcontroller’s power consumption (18.9 mW), and the clock frequency (64 MHz), the energy required for a single inference of the proposed model is calculated to be 5610.18 μJ, as shown in Equation (4). This value represents the total energy required by the MCU to complete one inference cycle, demonstrating its energy efficiency at handling the inference task. The proposed model is notably energy-efficient and ideal for long-term use in battery-operated devices.

(5) $$P_{\mathrm{sleep}}=V_{\mathrm{sleep}}\times I_{\mathrm{sleep}}$$

(6) $$\mathrm{Deployment}\;\mathrm{Duration}=\frac{\mathrm{Total}\;\mathrm{Battery}\;\mathrm{Energy}}{\mathrm{Total}\;\mathrm{Daily}\;\mathrm{Energy}\;\mathrm{Consumption}}$$

When in hibernation, the system consumes about 7.05 μW based on a hibernation current of 2.35 μA at 3 V and with no RAM retention during wakeup, as shown in Equation (5). Energy consumption varies across different inference models, as noted in reference [32]. Utilizing the power specifications for the proposed model network, the device can perform approximately 433,256.11 inferences with a standard 225 mAh coin cell battery. If the device operates one inference daily and remains in sleep mode otherwise, it could last for about 3956.88 days or nearly 11 years, as shown in Equation (6). This duration provides a foundational estimate of the energy requirements per inference cycle for each model, although actual energy use may vary depending on additional computations, peripheral activities, and specific neural network model workload characteristics, as mentioned in [60].

Overall, while individual model evaluations may show some limitations, the comprehensive performance, especially in resource-constrained conditions, emphasizes the distinct advantages and efficacy of the proposed model for edge computing applications.

## 5. Conclusions and Future Work

In this study, we proposed a specialized lightweight CNN model targeted for the structural health detection of supply canals. This model consists of an initial convolutional layer, two simplified inverted residual blocks, a global average pooling layer, and a dense layer containing multiple batch normalization and ReLU activation layers to enhance performance. The Adam learning algorithm is employed, and it achieves an accuracy of 94.17 ± 1.67% on the test set with a validation loss of 0.1976. Moreover, the lightweight model proposed in this paper has a parameter count of only 803 and a FLOP count of just 905,618, achieving high accuracy with minimal parameter count and complexity. It aims to optimize traditional detection methods and enhance both detection efficiency and accuracy. The proposed model is deployed for the first time on low-power, resource-constrained MCUs with the aim of supply canal structural health monitoring. An F1-score of 94.34 ± 1.64% is achieved with a model size of only 7.54 KB, an inference time of just 296.94 ms, flash memory usage of 354.48 KB, RAM usage of 96 KB, and power consumption per inference of only 5610.18 μJ. Deploying the model on low-power edge devices facilitates real-time monitoring and damage detection; data are processed and transmitted instantly, significantly improving the safety and maintenance strategies of water infrastructure [61].

The results of this study demonstrate that TinyML technology and lightweight models can effectively balance model performance with computational efficiency. The overall energy consumption of the system can be significantly reduced, thus extending the operating time of the device, which is especially important in battery-powered remote monitoring environments. This allows lightweight models to operate efficiently on resource-constrained equipment, enabling real-time monitoring and data processing. It provides a technical reference for more complex and comprehensive inspection equipment. Additionally, it offers a more efficient, cost-effective, and reliable solution for structural health monitoring of supply channels, contributing to the intelligence and autonomy of water infrastructure. Moreover, the use of remote sensing technology provides continuous large-scale geospatial data, which is crucial for monitoring large-scale water conservancy structures like supply canals. By integrating remote sensing data, structural health monitoring can be expanded to a wider area, enhancing both the coverage and richness of the data collected.

Despite significant advances in AI for predicting and diagnosing water infrastructure problems and monitoring structural health, challenges such as fluctuating model performance, dataset complexity, model initialization parameters, and training dynamics persist as barriers to practical deployment. In response to the lack of datasets, future efforts should incorporate lateral validation of the model’s generalization ability in various scenarios, such as downstream slopes in supply canals. Regarding model initialization parameters and training dynamics, advanced optimization algorithms and hyper-parameter tuning techniques, such as grid search or random search methods, can be employed to systematically explore different hyper-parameter combinations and identify the optimal model configuration.

Significant progress has been made in this study for classifying image-based damage using CNN. Nevertheless, numerous challenges persist in achieving safe and sustainable management of water infrastructure. For this reason, it is recommended that future research focuses on the following areas. Lateral validation of the model should be horizontally extended to mitigate the issue of an insufficient dataset—for example, to downstream slopes of water supply canals—in order to enhance generalization capabilities. Furthermore, while this study focused on categorizing damage, future efforts should integrate more layers of monitoring data, such as the temperature and humidity data from the Arduino Nano 33 BLE Sense development board used herein, in order to develop a more comprehensive condition detection system. Moreover, advanced data processing techniques, including data fusion methods, should be employed to integrate multiple data sources—image, temperature, and humidity—to offer more in-depth theoretical support and practical guidance.

## Figures and Tables

**Table 1:** Original dataset summary.

Category	Original Quantity	Train	Validation	Test
Normal	90	40	10	40
Crack	90	40	10	40
Hole	90	40	10	40
Total	270	120	30	120

**Table 2:** Augmented dataset and augmentation methods.

Category	Training	Validation	Test	Augmentation Method
Normal	240	60	40	Adjust contrast, image brightness, rotation, Gaussian noise
Crack	240	60	40	
Hole	240	60	40	
Total	720	180	120	

**Table 3:** Comparison of model performance.

Model	Parameters	FLOPs	Accuracy (%)	Precision (%)	Recall (%)	F1-Score (%)
ShuffleNetV2	1,194,515	20,652,799	94.95 ± 1.84	95.35 ± 1.82	95.09 ± 1.68	94.75 ± 1.70
ResNet-50	23,593,859	632,909,458	92.25 ± 1.97	92.78 ± 1.99	92.36 ± 1.66	92.33 ± 1.90
MobilenetV2	3,572,803	52,741,074	**96.75 ± 1.21**	**96.32 ± 1.56**	**96.02 ± 1.76**	**96.17 ± 1.67**
EfficientNet-B0	4,053,414	66,654,953	94.35 ± 2.19	94.55 ± 1.74	94.50 ± 2.00	95.11 ± 1.89
MnasNet	5,402,239	57,892,038	94.85 ± 1.84	94.33 ± 1.95	94.00 ± 2.10	94.05 ± 2.10
Our Model	**803**	**905,618**	94.17 ± 1.67	94.47 ± 1.46	94.27 ± 1.57	94.26 ± 1.94

**Table 4:** Microcontroller specifications for deployment.

Specification Category	Description
Microcontroller	nRF52840 (ARM Cortex-M4F 32-bit processor)
Clock Speed	64 MHz
CPU Flash Memory	1MB
Built-in Sensors	9-axis IMU (accelerometer, gyroscope, magnetometer), barometer, humidity sensor, temperature sensor, light sensor, and digital microphone
Dimensions	45 × 18 mm
Bluetooth	Bluetooth^®^ 5.0

**Table 5:** Performance and resource usage of deployed models.

Model	F1-Score (%)	Model Size (KB)	Inference Time (ms)	Flash (MB)	RAM (KB)	Power Consumption per Inference (μJ)
ShuffleNetV2	93.50 ± 1.88	4634.55	——	4.70	167.5	——
ResNet-50	92.56 ± 2.78	91,783.55	——	——	——	——
MobilenetV2	**96.63 ± 1.39**	13,795.55	——	14.10	826.8	——
EfficientNet-B0	94.42 ± 2.65	15,666.59	——	16.00	1200.0	——
MnasNet	94.32 ± 2.32	20,947.63	——	21.50	513.5	——
Our Model	94.34 ± 1.64	**7.54**	**296.94**	**0.35**	**96.0**	**5610.18**

## Data Availability

The data presented in this study are available on request from the corresponding author.
